# Transient penile erection following lumbar puncture with intrathecal prophylaxis in primary testicular diffuse large B-cell lymphoma: a case report

**DOI:** 10.3389/fonc.2025.1686603

**Published:** 2025-11-04

**Authors:** Tao Wang, Juan Yang, Hongbing Ma

**Affiliations:** Department of Hematology and Institute of Hematology, West China Hospital, Sichuan University, Chengdu, China

**Keywords:** primary testicular diffuse large B-cell lymphoma (PT-DLBCL), lumbar puncture (LP), intrathecal prophylaxis, penile erection, case report

## Abstract

Primary testicular diffuse large B-cell lymphoma (PT-DLBCL) is a rare and aggressive extranodal non-Hodgkin lymphoma that predominantly affects older men and carries a relatively high risk of central nervous system (CNS) relapse. Although lumbar puncture with intrathecal prophylaxis is commonly employed to reduce CNS involvement, transient penile erection after this procedure is extraordinarily rare. This case report describes a 51-year-old man diagnosed with PT-DLBCL (stage IA) who underwent right radical orchiectomy, followed by six cycles of R2-CHOP chemotherapy with intrathecal prophylaxis prior to each cycle. Approximately 30 minutes after the sixth lumbar puncture, the patient developed a transient penile erection that persisted for approximately three hours before resolving spontaneously without intervention. He later received additional cycles of R-HD-MTX and scrotal radiotherapy, and no further episodes of penile erection or disease recurrence were observed. This clinical vignette highlights the potential for rare neurogenic reflex erections associated with repeated lumbar punctures, underscoring the importance of heightened vigilance, procedural refinement, and close monitoring in patients receiving multiple intrathecal treatments.

## Introduction

Primary testicular diffuse large B-cell lymphoma (PT-DLBCL) is a rare extranodal non-Hodgkin lymphoma accounting for approximately 10% of all testicular malignancies but only 1–2% of all non-Hodgkin lymphomas (1, 2). PT-DLBCL typically occurs in older men, with a median onset age of 67 years and a relatively poor prognosis (2). Although its incidence is low, PT-DLBCL is the most common type of testicular malignancy in elderly men and one of the most frequently reported primary extranodal lymphomas in this population (3). PT-DLBCL patients exhibit distinct clinical features and biological behaviors, including a greater risk of CNS relapse and relative resistance to conventional chemotherapy regimens (1, 4).

The current standard treatment strategy for PT-DLBCL includes ipsilateral orchiectomy followed by immunochemotherapy with rituximab and anthracycline agents (e.g., R-CHOP), prophylactic radiotherapy to the contralateral testis, and CNS prophylaxis (5). Lumbar puncture with intrathecal chemotherapy is the most commonly used approach to reduce CNS relapse (6). However, these interventions may lead to various complications. Among them, short-lived penile erection following lumbar puncture is extremely rare in the medical literature (7).

Here, we report the case of a 51-year-old man with PT-DLBCL who experienced transient penile erection after routine lumbar puncture and intrathecal chemotherapy. On the basis of the clinical course and management, we highlight that this rare complication is potentially associated with lumbar puncture. The underlying pathophysiological mechanisms and management strategies are also discussed.

## Case report

A 51-year-old man (height 160 cm, weight 50 kg, body mass index 19.5 kg/m²) presented with a 20-day history of mild discomfort in the right testis. Physical examination revealed enlargement of the right testis (approximately 4 × 3 × 2.5 cm), with a firm texture, limited mobility, and indistinct borders between the testis and epididymis. The scrotal skin and temperature were normal, without tenderness, and the transillumination test was negative. Initial laboratory tests (complete blood count, serum biochemistry, coagulation function) revealed no significant abnormalities, with no evidence of systemic disease. Scrotal ultrasound revealed an enlarged prostate, parenchymal echo changes in the right testis with increased blood flow signals, and a right hydrocele. Plain and contrast-enhanced pelvic computed tomography (CT) revealed prostatic hyperplasia and an enlarged right testis with heterogeneous hyperintensity on enhancement, along with right spermatic vein varicosities and bilateral slight hydrocele, suggesting a neoplastic lesion.

Under combined spinal–epidural anesthesia, the patient underwent a right radical orchiectomy. Pathological examination of the surgical specimen revealed invasive B-cell lymphoma. Immunohistochemistry revealed positivity for CD20 and CD79a, as well as Ki-67 positivity, in approximately 80% of the tumor cells, whereas CD3, CD30, CK (Pan), SALL4, PLAP, S100, and Desmin were negative. These findings confirmed the diagnosis of diffuse large B-cell lymphoma (DLBCL). Postoperative evaluation, including bone marrow aspiration, flow cytometry, and biopsy, revealed no abnormalities. Whole-body positron emission tomography–computed tomography (PET-CT) revealed no distant lesions. The patient was diagnosed with PT-DLBCL, stage IA. He underwent six cycles of R2-CHOP chemotherapy, comprising rituximab (600 mg, Day 1), cyclophosphamide (1100 mg, Day 1), vincristine (4 mg, Day 1), prednisone (50 mg bid, Days 1–5), and lenalidomide (25 mg qd, Days 1–10). After the fourth cycle, PET-CT revealed no evidence of lymphoma. Before each chemotherapy cycle, a lumbar puncture with intrathecal prophylaxis (dexamethasone 5 mg, methotrexate 10 mg, and cytarabine 30 mg) was performed at the L3–L4 interspace via a 22-gauge spinal needle, following local anesthesia with 5 mL of 2% lidocaine. Routine, biochemical, and flow cytometric analyses of cerebrospinal fluid (CSF) revealed no abnormalities throughout treatment.

Notably, approximately 30 minutes after the sixth lumbar puncture, the patient experienced a transient episode of penile erection while lying supine. He reported no pain, discomfort, backache, or lower-limb numbness. The erection resolved spontaneously after approximately three hours without any intervention. The patient thereafter completed two additional cycles of R-HD-MTX with intrathecal therapy, during which no further episodes of penile erection occurred. Subsequent scrotal radiotherapy (25 Gy) was completed in September 2024. During the follow-up period, the patient’s testosterone level and sexual function remained normal. He continues regular CT evaluations with no evidence of disease recurrence.

## Discussion

PT-DLBCL has a pronounced tendency to involve the central nervous system, so contemporary treatment protocols routinely incorporate lumbar intrathecal chemotherapy to mitigate this risk (8, 9). Aside from postdural-puncture headache and short-lived radicular irritation, lumbar puncture itself is generally viewed as a low-risk procedure, with grade ≥ 2 adverse events reported in fewer than 2% of cases (10). Even so, our patient developed a transient, painless penile erection after his sixth puncture—an event described only sporadically following spinal or epidural anesthesia and, to our knowledge, never before in the setting of prophylactic intrathecal therapy for lymphoma (11, 12). Because rigidity subsides spontaneously within three hours—below the 4-hour threshold that defines priapism—and is unaccompanied by dysuria or pain, classical low-flow priapism is confidently excluded (13, 14).

**Figure 1:**
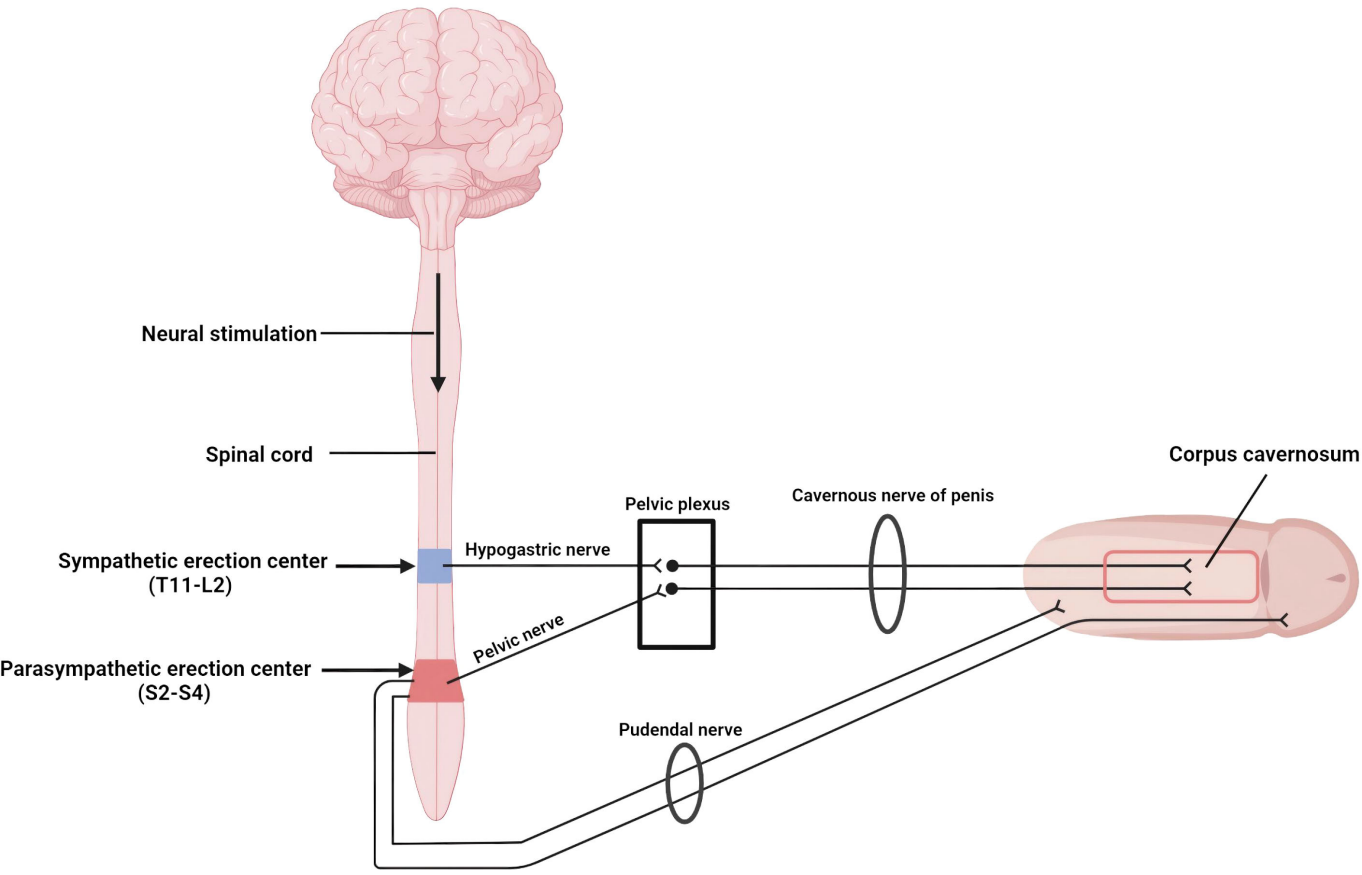
Neural pathways mediating reflex erection: Sympathetic fibers from T11mrs keep the penis flaccid, whereas parasympathetic fibers from S2omr trigger erection. Image created with BioRender.com.

Physiologically, erection is coordinated by two spinal centers that receive supraspinal modulation (**Figure 1**). Continuous output from the thoracolumbar sympathetic center (T11–L2) traveling through the hypogastric plexus maintains flaccidity, whereas activation of the sacral parasympathetic center (S2–S4) sends pelvic splanchnic efferents that initiate tumescence (15, 16). Somatic fibers arising in Onuf’s nucleus run within the pudendal nerve to the perineal muscles, providing additional rigidity. Sensory volleys conveyed by the dorsal penile and perineal nerves can therefore trigger a purely segmental reflex erection when they reach the sacral cord in the setting of diminished sympathetic counterbalance (17).

We believe that the transient erection in this case represents a benign sacral reflex precipitated by temporary sympathetic blockade produced by lidocaine used for local infiltration before dural puncture. The patient’s lean habitus (BMI 19.5 kg/m²) offered minimal adipose buffering, facilitating anesthetic diffusion along tissue planes. Critically, although the same interspace (L3–L4), needle gauge, lidocaine dose (5 mL of 2%), and patient position were maintained across all six procedures, the sixth puncture was performed by a different operator. Subtle technical variations—such as the needle trajectory angle, depth of subcutaneous infiltration, or injection speed—may have altered the anesthetic distribution pattern, allowing lidocaine to track more readily toward the thoracolumbar sympathetic chain (T11–L2) within the ensuing 20–30 minutes. Small-diameter sympathetic fibers possess a low safety margin for sodium channel conduction and are selectively susceptible to lidocaine; their inhibition removes the tonic anti-erectile signal, allowing unopposed parasympathetic outflow and generating a reflex erection. This phenomenon abated as the local lidocaine concentration waned, in keeping with the drugin approximately 90-minute intrathecal half-life and a clinical effect that seldom persists beyond 2–3 hours (18).

Neural susceptibility may have been heightened by prior intrathecal chemotherapy. Methotrexate and cytarabine can provoke low-grade chemical meningitis characterized by perineural edema and cytokine release (19, 20). This cumulative neurotoxicity is supported by clinical evidence: a prospective study in pediatric leukemia patients demonstrated increased rates of headache and vomiting alongside cerebrospinal fluid chemical alterations after repeated intrathecal injections (21), whereas a systematic review confirmed that ≥5 intrathecal procedures increase the risk of severe complications such as ascending paralysis (22). In the present case, although the manifestation was benign and self-limiting, its occurrence after the sixth lumbar puncture may suggest a similar cumulative sensitization effect. Inflammatory priming of the dorsal roots lowers the threshold for lidocaine-induced conduction block, so a standard dose of anesthetic might more easily silence sympathetic fibers after multiple prophylactic injections. In addition, brief procedural discomfort, a momentary Valsalva maneuver (23), or anxiety-induced sympathetic withdrawal might have further shifted the autonomic balance toward parasympathetic predominance, thereby amplifying the response.

Management in similar scenarios is essentially conservative. Provided that detumescence occurs within two to three hours and that the patient remains comfortable, observation and reassurance are adequate. Persistence beyond four hours, escalating pain, or voiding difficulty should prompt the transition to the conventional priapism algorithm, including intracavernosal phenylephrine, corporal aspiration, or surgical shunting, as dictated by urological guidelines (13). In our patient, no intervention was necessary, and no recurrence was documented during subsequent high-dose methotrexate cycles. To mitigate the risk of recurrence, several procedural refinements can be implemented. Precise needle placement should be verified at, or preferably distal to, the L3–L4 interspace. Lidocaine must be restricted to the minimal effective dose, and the local anesthetic should be injected slowly and in incremental aliquots to curb cephalad diffusion along fascial planes. When feasible, puncture should be performed under continuous real-time ultrasonographic guidance (24).

Several limitations merit acknowledgment. Although our proposed mechanism of lidocaine-mediated sympathetic blockade is physiologically plausible, it remains largely inferential in the absence of direct evidence. Given the self-limiting nature of the symptoms, further diagnostic investigations, such as ultrasound or MRI, were not pursued. Consequently, alternative or contributory mechanisms cannot be categorically excluded. Direct mechanical stimulation of sacral nerve roots during needle advancement, transient psychological arousal, or systemic absorption of lidocaine may play ancillary roles. Notably, intrathecal opioid administration has been reported to induce priapism (25), suggesting that this phenomenon may not be specific to lidocaine. Furthermore, as a single case report, the clinical significance of this observation remains limited, and broader conclusions regarding incidence or risk factors cannot be drawn.

In conclusion, transient penile erection after lumbar puncture with intrathecal prophylaxis appears to reflect a benign sacral reflex triggered by temporary sympathetic blockade from cephalad-diffusing lidocaine in a sensitized neural milieu. Awareness of this mechanism enables clinicians to counsel patients appropriately, avoid unnecessary intervention, and adjust techniques for individuals requiring repeated intrathecal therapy.

## Data Availability

The datasets presented in this article are not readily available because of ethical and privacy restrictions. Requests to access the datasets should be directed to the corresponding author.
